# The role of acidification in the inhibition of *Neisseria gonorrhoeae *by vaginal lactobacilli during anaerobic growth

**DOI:** 10.1186/1476-0711-10-8

**Published:** 2011-02-17

**Authors:** Michelle A Graver, Jeremy J Wade

**Affiliations:** 1 Medical Microbiology, King's College Hospital, Denmark Hill, London, SE5 9RS, UK

## Abstract

**Background:**

Vaginal lactobacilli protect the female genital tract by producing lactic acid, bacteriocins, hydrogen peroxide or a local immune response. In bacterial vaginosis, normal lactobacilli are replaced by an anaerobic flora and this may increase susceptibility to *Neisseria gonorrhoeae*, a facultative anaerobe. Bacterial interference between vaginal lactobacilli and *N. gonorrhoeae *has not been studied in liquid medium under anaerobic conditions. By co-cultivating *N. gonorrhoeae *in the presence of lactobacilli we sought to identify the relative contributions of acidification and hydrogen peroxide production to any growth inhibition of *N. gonorrhoeae*.

**Methods:**

Three strains of *N. gonorrhoeae *distinguishable by auxotyping were grown in the presence of high concentrations (10^7^-10^8 ^cfu/mL) of three vaginal lactobacilli (*L. crispatus*, *L. gasseri *and *L. jensenii*) in an anerobic liquid medium with and without 2-(*N*-morpholino)-ethanesulfonic (MES) buffer. *Fusobacterium nucleatum *was used as an indicator of anaerobiosis. Bacterial counts were performed at 15, 20 and 25 h; at 25 h pH and hydrogen peroxide concentrations were measured.

**Results:**

Growth of *F. nucleatum *to >10^8 ^cfu/mL at 25 h confirmed anaerobiosis. All bacteria grew in the anaerobic liquid medium and the addition of MES buffer had negligible effect on growth. *L. crispatus *and *L. gasseri *produced significant acidification and a corresponding reduction in growth of *N. gonorrhoeae*. This inhibition was abrogated by the addition of MES. *L. jensenii *produced less acidification and did not inhibit *N. gonorrhoeae*. Hydrogen peroxide was not detected in any experiment.

**Conclusions:**

During anaerobic growth, inhibition of *N. gonorrhoeae *by the vaginal lactobacilli tested was primarily due to acidification and abrogated by the presence of a buffer. There was no evidence of a specific mechanism of inhibition other than acid production under these conditions and, in particular, hydrogen peroxide was not produced. The acidification potential of vaginal lactobacilli under anaerobic conditions may be their most important characteristic conferring protection against *N. gonorrhoeae *infection.

## Background

The majority of healthy women harbor lactobacilli (LB) in the vagina at counts of 10^7^-10^9 ^cfu/gm secretions [1,2]. These protect the female genital tract by metabolising glycogen in shed epithelial cells to produce lactic acid, thereby generating a low pH environment. Most vaginal lactic acid is of the D-lactate isomer and therefore of bacterial, not human, origin. LB may also inhibit opportunist bacteria through production of bacteriocins, hydrogen peroxide (H_2_O_2_) or local immune stimulation, as well as through nutrient and stearic competition.

The hallmark of bacterial vaginosis (BV) - a highly prevalent condition of global public health importance - is replacement of normal vaginal LB by a mixed flora of anaerobic bacteria. Studies suggest that an abnormal vaginal flora increases susceptibility to infection with sexually-transmitted pathogens including *Neisseria gonorrhoeae *(NG).

Evidence accrues that NG grows anaerobically *in vivo: *NG may be recovered with obligate anaerobes from clinical material and NG proteins induced anaerobically include a nitrite reductase, antibodies to which are present in sera of infected patients [3,4]. Bacterial interference between LB and NG growing anaerobically in liquid medium has not been studied. By co-cultivating NG and LB we sought to identify the relative contributions of LB acidification and H_2_O_2 _production to any inhibition of NG under anaerobic conditions.

## Methods

### Media

A defined medium [5] rendered anaerobic with 'Oxyrase for Broth' (Oxyrase Inc, Ohio, USA) at 20 mL/L [6] was used, with and without 30 mM 2-(*N*-morpholino)-ethanesulfonic acid (MES) buffer adjusted to an initial pH 6.5 with 1 M HCl. The medium was sterilized through a 0.22 μ filter (Nalgene) and 7 mL volumes added to 7 mL screw-top plastic bijoux (Sterilin) giving a headspace above the liquid in each bijoux of approximately 1 mL. Media were pre-reduced before inoculation by incubating at 37°C for ≥2 h. Bijoux containing medium with rezasurin sodium at 0.002 g/L were included as uninoculated reduction controls in each experiment.

### Bacterial strains

The strains studied included three LB: *Lactobacillus crispatus *NCTC 4505 (LC), *Lactobacillus gasseri *ATCC 9857 (LG) and *Lactobacillus jensenii *ATCC 25258 (LJ). Modified tetramethylbenzidine (TMB) medium, prepared and used as described by Rabe and Hillier [7], revealed that the LJ, LC and LG were strong, weak and negative H_2_O_2 _producers, respectively. Three clinical isolates of NG distinguishable by auxotyping by the method of Copley & Egglestone [8] were studied. In each experiment *Fusobacterium nucleatum *ATCC 25586 (FN) was included as an indicator of anaerobiosis.

### Preparation of inocula

Inocula were prepared from overnight growth on chocolate agar in 5% CO_2 _at 37°C for NG, from blood agar incubated anaerobically overnight at 37°C for FN, and from blood agar incubated in 5% CO_2 _at 37°C for 48 h for LB (media from Oxoid, Basingstoke, UK).

### Monomicrobial culture

Monomicrobial growth studies were done to ensure that all bacteria were capable of growth in the medium, to identify any effect of MES buffer on growth, and to confirm anaerobiosis by growth of the anaerobe FN. Bacteria were suspended in phosphate-buffered saline (PBS) and suspension added to media to give an inoculum of 10^4^-10^5 ^cfu/mL for monomicrobial growth studies for all bacteria.

### Co-cultivation

Co-cultivation experiments were designed to demonstrate whether high concentrations of LB affect the growth of NG and if the addition of a pH buffer had any influence. In co-cultivation experiments, the growth of each NG in the presence of each LB was assessed by adding an inoculum of 10^4^-10^5 ^cfu/mL of each NG (prepared as above) to a high inoculum of each LB. This high inoculum, of 10^6^-10^7 ^cfu/mL, was achieved by preparing a suspension of LB in PBS before adding to the media; monomicrobial growth curves were also done for the LB using this higher inoculum.

### Sampling, colony counts and growth curves

Capped bijoux were incubated at 37°C, and at times 15 h, 20 h and 25 h resazurin indicator controls were examined and confirmed reduction at inoculation and up to at least 25 h. At the same time intervals, counts were done after vortexing for 5 s, on serial 10^-2 ^dilutions in PBS on chocolate agar for NG and blood agar for LB and FN using a spiral plater (Don Whitley, UK). At 25 h pH was measured using a Mettler Toledo 'SevenEasy' pH meter, and H_2_O_2 _assayed by pipetting 50 μL of medium on to Quantofix Peroxide 100 test strips and recording the colour change as per the manufacturer's instructions (Macherey-Nagel GmbH & Co KG, Germany). These strips are capable of detecting 1-100 mg/L peroxide. All plates were incubated at 37°C for 48 - 72 h: the chocolate agar for NG and blood agar for LB plates in 5% CO_2_, and blood agar for FN in anaerobic jars. Colonies were counted using an aCOLyte colony counter (Don Whitley, UK). All experiments were in triplicate. To demonstrate growth in the media, and any effect of MES buffer, growth curves were prepared for the FN, the three NG, and the three LB (for the latter, from both low and high inocula), with and without MES, using the median bacterial count at each time point. Similarly, in co-cultivation experiments growth curves for the three NG in the presence of each of the LB, with and without MES buffer, were prepared by plotting median values.

## Results

**Figure 1:**
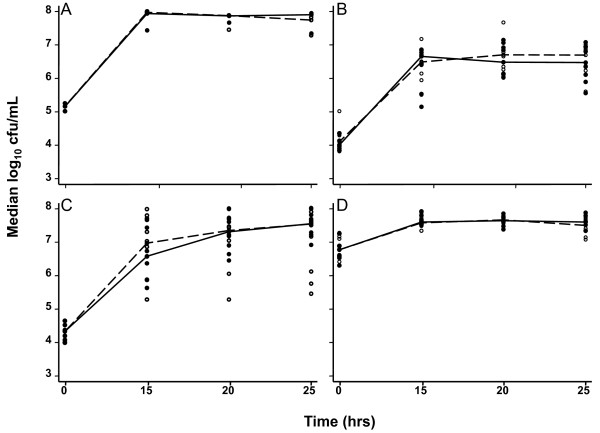
**Monomicrobial growth curves**. Growth curves with (—) and without (--) MES for *F. nucleatum *(A), three strains of *N. gonorrhoeae *(B), and three lactobacilli from low inocula (C) and from the high inocula used in co-cultivation experiments (D). Lines connect median values for all experiments.

The median counts (median log_10 _cfu/mL) of triplicates for the obligate anaerobe FN at times 0 and 25 h were 5.27 and 8.2, respectively, confirming anaerobiosis (Figure 1A). All LB and NG grew in media with and without MES buffer in the monomicrobial growth studies (Figure 1B and 1C). LB at the high inoculum used in co-cultivation experiments grew over the first 15h after inoculation and remained viable thereafter (Figure 1D). Throughout, the addition of MES had negligible effect on growth.

**Figure 2:**
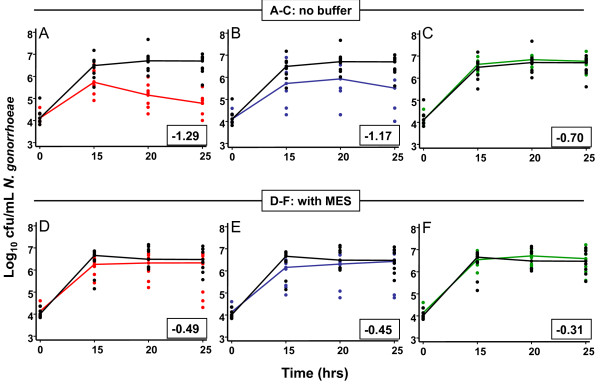
**Growth curves for *N. gonorrhoeae***. Growth of *N. gonorrhoeae *alone (black lines; as in Figure 1B) and in the presence of high inoculum of lactobacilli: *L. crispatus *(red lines; A and C), *L. gasseri *(blue lines; B and E), or *L. jensenii *(green lines; C and F), with (A-C) and without (D-F) MES buffer. Lines connect median value for triplicate experiments. Numbers in boxes above X axis show change in pH units of medium containing lactobacilli between times 0 and 25 h.

In co-cultivation experiments, the median log_10_cfu/mL inocula for LB and NG were 6.78 and 4.0, respectively (Figure 1D & Figure 2). In the absence of MES buffer, both LC and LG reduced the pH by >1 pH point by 25 h; MES resisted this acidification, with a pH fall of <0.5 pH value for both. In contrast, LJ reduced the pH by only 0.7 pH point with a correspondingly lower pH fall in the presence of MES. Although the three NG grew in the presence of a high concentration of each LB, NG growth with LC and LG was reduced compared to growth without LB, and MES abrogated this inhibition. The presence of LJ had no effect on NG whether MES was included or not. H_2_O_2 _was not detected at 25 h in any experiment.

## Discussion

The LB studied here include representatives of the three species most frequently recovered from the healthy pre-menopausal vagina: *L. crispatus*, *L. gasseri *and *L. jensenii*. The normal vaginal flora plays a key role in maintaining vaginal health and, compared to the flora at other mucosal surfaces, is relatively simple in health: most women are colonized by a predominant species of LB. BV is a common condition - with an incidence of approximately 20 - 30% of adult women - characterized by loss of the protective LB flora in the vagina. BV is an important public health problem as it is a risk factor for obstetric complications and the sexually-transmitted infections gonorrhoea, *Chlamydia trachomatis *and *Trichomonas vaginalis *[9-13]. The normal vagina is anaerobic with pressures of O_2 _and CO_2 _lower and higher, respectively, than atmospheric levels, though menstruation increases oxygen tension [14]. In BV, LB are replaced by a mixture of anaerobic bacteria and antimicrobials with activity against anaerobes, such as metronidazole and clindamycin, are the mainstay of treatment.

Hydrogen peroxide production is considered an important protective characteristic of LB comprising the normal vaginal flora. TMB medium, originally described by Eschenbach *et al *[15] and optimized by Rabe & Hillier [7], demonstrates the ability of a LB to produce H_2_O_2 _when colonies grown anaerobically on the medium are subsequently exposed to air. Although there is an association between H_2_O_2 _production and a stable, and therefore protective, vaginal LB flora, it is unclear whether LB produce H_2_O_2 _under the low oxygen tensions found in either the normal or BV vagina [16,17]. The need for agitation of cultures to generate detectable H_2_O_2 _has been described before [16]. It is feasible that LB capable of producing H_2_O_2 _do so for self-preservation when oxygen levels increase during menses. The antibacterial effects of H_2_O_2_, either directly or in concert with halide ions and myeloperoxidases [18], may permit LB to suppress other bacteria and thereby control the vaginal flora. This would reconcile the findings of studies that emphasize the importance of H_2_O_2_-producing LB for vaginal health with the anaerobic conditions found in the vagina.

We are not aware of another study of bacterial interference between LB and NG during anaerobic co-cultivation. Interactions between LB and NG have been sought using agar overlay methods [17,19-21] or by brief co-incubation [22] under aerobic conditions. Our results confirm that NG can grow under strictly anaerobic conditions and may be inhibited by vaginal LB growing under conditions more akin to those seen in the healthy or BV vagina than those previously used in for bacterial interference studies. This inhibitory action appears to be primarily due to acidification rather than H_2_O_2 _production as demonstrated by the correlation between the degree of inhibition and the acidification capabilities of individual LB, the effects of buffer, and lack of H_2_O_2 _production. It remains possible, however, that a bacteriocin produced at, or activated by, a lower pH could account for some of our findings. These observations suggest that those seeking to select LB for probiotic use in BV should address the acidification potential of candidate LB.

## Conclusions

The normal vagina is anaerobic with pressures of O_2 _and CO_2 _lower and higher, respectively, than atmospheric levels. During anaerobic growth, inhibition of *N. gonorrhoeae *by the vaginal LB tested was primarily due to acidification, as evidenced by reduced inhibition is the presence of buffer. Under these conditions H_2_O_2 _was not produced by LB shown to be capable of H_2_O_2 _production using standard methods. The acidification potential of vaginal lactobacilli under anaerobic conditions may be an important characteristic conferring protection against NG infection.

## Abbreviations used

BV: bacterial vaginosis; Cfu: colony-forming unit; FN: *Fusobacterium nucleatum; *H_2_O_2_: hydrogen peroxide; L: litre; LB: lactobacilli; LC: *Lactobacillus crispatus; *LG: *Lactobacillus gasseri; *LJ: *Lactobacillus jensenii; *mL: millilitre; NG: *Neisseria gonorrhoeae; *PBS: phosphate-buffered saline.

## Competing interests

The authors declare that they have no competing interests.

## Authors' contributions

Both authors contributed equally to the design and execution of experiments and preparation of the manuscript. Both authors have read and approved the final manuscript.
